# Absence of neural speech discrimination in preterm infants at term-equivalent age

**DOI:** 10.1016/j.dcn.2019.100679

**Published:** 2019-07-10

**Authors:** Lisa Bartha-Doering, Johanna Alexopoulos, Vito Giordano, Lisa Stelzer, Theresa Kainz, Silvia Benavides-Varela, Isabell Wartenburger, Katrin Klebermass-Schrehof, Monika Olischar, Rainer Seidl, Angelika Berger

**Affiliations:** a Department of Pediatrics and Adolescent Medicine, Medical University Vienna, Vienna, Austria; b Comprehensive Center for Pediatrics, Medical University of Vienna, Vienna, Austria; c Department of Psychoanalysis and Psychotherapy, Medical University Vienna, Vienna, Austria; d Department of Developmental Psychology and Socialization, University of Padova, Padova, Italy; e Cognitive Sciences, Department of Linguistics, University of Potsdam, Potsdam, Germany

**Keywords:** Near-infrared spectroscopy, Preterm birth, Newborn infants, Language development, Speech discrimination

## Abstract

Children born preterm are at higher risk to develop language deficits. Auditory speech discrimination deficits may be early signs for language developmental problems. The present study used functional near-infrared spectroscopy to investigate neural speech discrimination in 15 preterm infants at term-equivalent age compared to 15 full term neonates. The full term group revealed a significantly greater hemodynamic response to forward compared to backward speech within the left hemisphere extending from superior temporal to inferior parietal and middle and inferior frontal areas. In contrast, the preterm group did not show differences in their hemodynamic responses during forward versus backward speech, thus, they did not discriminate speech from non-speech. Groups differed significantly in their responses to forward speech, whereas they did not differ in their responses to backward speech. The significant differences between groups point to an altered development of the functional network underlying language acquisition in preterm infants as early as in term-equivalent age.

## 1 Introduction

Worldwide, 11% of all live births are born preterm (Blencowe et al., 2012). Children born prematurely are prone to health consequences far beyond the first months of live. They are not only at greater risk for persistent neurological disorders including sensory and motor impairments; up to half of all preterm born infants develop cognitive deficits that influence their later academic achievement (Allotey et al., 2017; Twilhaar et al., 2018).

Language deficits are among the most commonly reported neurodevelopmental deficits in preterm born children (Barre et al., 2011). Studies have identified significant delays in the development of expressive language functions, which manifest themselves in poor grammatical and vocabulary skills (Arpino et al., 2010; Guarini et al., 2016; Taylor et al., 2013; Vohr, 2014). After school entry, preterm-born children frequently show poor reading and writing acquisition (Guarini et al., 2009; Wolke et al., 2008). These early deficits often result in persistent impairments in grammatical skills and literacy (Guarini et al., 2010; van Noort-van der Spek et al., 2012). Language deficits furthermore contribute to the poor socio-emotional development often observed in preterm born children and thus influence their relationships to friends and reduce their quality of life (Gire et al., 2019; Montagna and Nosarti, 2016).

Infant studies have shown that early discrimination of auditory input predict later language skills during language acquisition (Benasich and Tallal, 2002; Kuhl and Rivera-Gaxiola, 2008). Phonological discrimination in infants is related to later literacy skills (Schaadt et al., 2015; van Zuijen et al., 2013), and prosodic discrimination in six months old infants predicts vocabulary growth (Cristia and Seidl, 2011). Full-term neonates are able to discriminate intonation and speech sounds such as isolated vowels (Kujala et al., 2004; Nazzi et al., 1998; Ramus et al., 1999). In infants born preterm, studies have reported early deficits in prosodic discrimination (Herold et al., 2008), in phonological discrimination (Peña et al., 2010a), and in discrimination of rhythmically similar languages (Peña et al., 2010b). However, little information is available about neural speech discrimination in preterm infants and potential differences when compared to full term neonates. Mahmoudzadeh and coworkers investigated cerebral responses to syllables in very preterm infants of 28–32 weeks of gestational age (GA) and demonstrated discrimination responses to phoneme changes (/ba/ vs. /ga/) in bilateral temporal and inferior frontal regions (Mahmoudzadeh et al., 2013, 2017). Very recently, Arimitsu et al. (2018) reported gestational differences in the typicality of hemodynamic response function (increased oxyhemoglobin) in response to phonetic changes (/itta/ vs. /itte/) and prosodic changes (/itta/ vs. /itta?/) of speech in preterm infants. They showed that preterm neonates exhibited atypical response patterns and atypical lateralization in response to speech changes when examined before 39 weeks of GA. However, these atypical patterns in terms of lateralization and direction of hemodynamic repsonses diminished when preterms reached term age. Yet, while preterm born infants showed no categorial differences in the “typicality of hemodynamic response function” at term anymore, it remains unclear if they still exhibited quantitative differences in their hemodynamic responses compared to full term infants. Overall, previous studies point to the existence of basic phoneme discrimination in preterm infants at term-equivalent age. It remains unclear, however, whether neural discrimination differs between preterm infants at term-equivalent age and full term neonates.

Previous studies of preterm brain development show structural alterations in brain structures relevant to language compared to full term born peers (Frye et al., 2010; Mullen et al., 2011; Reidy et al., 2013) and suggest that preterm born adolescents rely upon different white-matter pathways for the development of language, with the degree of this alteration being directly related to cognitive performance (Myers et al., 2010; Salvan et al., 2017; Schafer et al., 2009). Studies including older children indicate that the language network in children born preterm develops differently compared to term born children (Barde et al., 2012; Peterson et al., 2003). Moreover, altered intra- and inter-hemispheric connectivity between language and non-language regions has been found in preterm-born children (Gozzo et al., 2009; Northam et al., 2012; Wilke et al., 2014). However, whether the differences in the functional language network between preterm and full term born children are already present at birth, or whether they are the effect of altered language acquisition and processing in former preterm born children, still remains unclear.

Language develops in stages that build on one another, and a deficit in one stage will potentially delay the next (Guzzetta, 2014; Kuhl, 2004). Early detection of auditory language discrimination deficits enhances early intervention possibilities and thus increases the chance to ameliorate language developmental delays (Guzzetta et al., 2011). In preterm birth, these therapeutic interventions may start within the first months of age, when neuroplasticity mechanisms are thought to be greatest (Fiori and Guzzetta, 2015; Martinez-Biarge et al., 2010). However, to date, the existence of neurobiological markers of later language deficits in single individuals at birth are not yet proved. The present study therefore investigated possible differences in neural speech discrimination and the functional language network underlying speech discrimination in preterm infants at term-equivalent age compared to full term neonates. We used functional near-infrared spectroscopy (fNIRS) to test neural discrimination of speech forward versus speech backward and hypothesized reduced neural speech discrimination and differences in the underlying developing language network of preterm infants at term-equivalent age compared to full term neonates.

## 2 Methods

### 2.1 Study participants

Fifteen infants born preterm (25 to 36 weeks of gestation) and 15 neonates born at term age (38 to 41 weeks) were included in the present study. Seven further infants (three preterm infants) had to be excluded from the study due to excessive motion-related artifacts during fNIRS measurement. Preterm infants were measured around term-equivalent age, a few days before discharge; full term neonates were investigated 1 to 3 days after birth. Table 1 contains information about participants’ characteristics at birth and at test (for term neonates, head circumferences and weight measurements at test were taken from the day of birth). At birth, groups significantly differed in their GA, weight, head circumference, and APGAR score (p < .007 for all comparisons). At test, groups did not significantly differ in GA, head circumference, or weight.

**Table 1:** Sample characteristics.

	Preterm infants mean ± SD (range)	Term infants mean ± SD (range)	*P* value
Participants, n	15	15	
Sex (female, male)	6/9	9/6	.367
**At birth:**			
GA at birth (wk)	29.45 ± 3.57 (25.00 – 36.14)	38.70 ± 1.15 (37.43 – 40.43)	.000*
HC at birth (cm)	27.29 ± 4.03 (21.80 – 35.00)	35.67 ± 4.57 (30.50 – 51.00)	.000*
Weight at birth (g)	1087 ± 498 (350 – 2160)	3129 ± 462 (2320 – 3890)	.000*
Apgar at minute 10	9.13± .35 (9 – 10)	9.67 ± .62 (8 – 10)	.007*
**At test:**			
GA at test (wk)	38.38 ± 1.76 (36.14 – 41.72)	39.01 ± 1.11 (37.57 – 40.58)	.125
HC at test (cm)	33.86 ± 1.52 (31.00 – 37.50)	35.67 ± 4.57 (30.50 – 51.00)	.158
Weight at test (g)	2651 ± 506 (1900 – 3544)	3129 ± 462 (2320 – 3890)	.307

HC, head circumference; GA, gestational age.

* Indicates significance after FDR correction.

All children were born at the Department of Neonatology at the Medical University of Vienna. Inclusion criteria were a) normal auditory evaluation as measured by auditory brainstem response; b) normal neurological findings including normal clinical examination and normal ultrasound scan; d) both parents native speakers of German; e) both parents right-handed; f) no reported language- or reading related problems for either parent. Exclusion criteria were congenital malformations and known chromosomal abnormalities. Written informed consent was obtained from a parent in all children. This study was approved by the Ethics Committee of the Medical University Vienna and was conducted in accordance with the Helsinki Declaration of 1975.

### 2.2 Stimuli

The speech discrimination paradigm was adapted from Peña et al. (2003) who used normal speech stimuli compared to backward speech or silence to test language localization in infants. In full term infants, forward speech has shown to elicit robust temporal and frontal activations (Dehaene-Lambertz et al., 2002; Peña et al., 2003; Sato et al., 2012). In the present study, speech samples of a female speaker were recorded while she recited a children’s story using infant-direct speech (Lobe and Weigel, 1972). Stories were edited to ten sequences of 15 s with well-formed and complete prosodic units each (mean pitch 233 Hz). Mean intensity of sentences was equalized (mean intensity 70 dB). Each sequence was time-backwarded using version 2.1.2 of Audacity(R) recording and editing software (Audacity, 2012), thus generating backward speech (= non-speech) stimuli with the same acoustic and phonetic features, but with distorted phonological, semantic, and prosodic information. This resulted in 20 sequences overall. Each sequence was followed by silence with randomized length (10–15 s). The order of sequences was pseudo-randomized with not more than two consecutive sequences of the same condition, and counterbalanced across participants. Overall duration of the fNIRS paradigm was 9 min 10 s.

### 2.3 Procedure

**Fig. 1:**
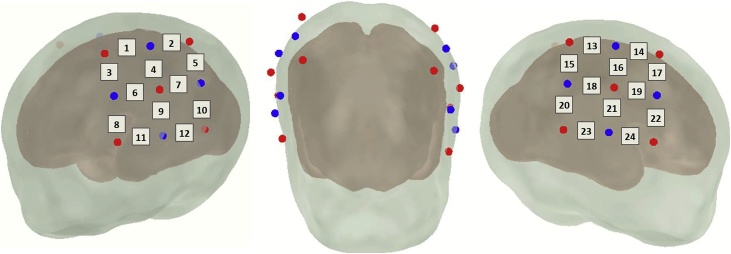
Optode placement overlaid on a schematic newborn head. Red dots indicate sources, blue dots indicate detectors, and numbers correspond to measurement channels (For interpretation of the references to colour in this figure legend, the reader is referred to the web version of this article).

A Hitachi ETG-4000 NIRS machine was used with 24 optical channels. Separation between emitters and detectors was 2 cm. The total laser power was set at 0.75 mW, and two continuous light sources used 695 nm and 830 nm wavelengths were used. The optical fibers were embedded in soft silicon cushions of two light-weight probes designed for use with neonates (Hitachi Neonate Probes). These probes were placed directly above the ear using the bilateral preauricular points as the reference to align the bottom finger of the probe (channels 3, 6, 8, and 11 in the left hemisphere; channels 17, 19, 22, and 24 in the right hemisphere) with the temporal areas (T3 to T5 and T4 to T6 lines in the left and right hemispheres, respectively). Thereafter, the fibers exiting the silicon holders were oriented in such a way that they crossed symmetrically above the glabella (midpoint between the eyebrows) in the center of the forehead (Fig. 1).

Infants were tested in a quiet, dimly lit room within the Department of Neonatology, Medical University of Vienna, directly after feeding, lying in their cribs with their eyes closed in a state of rest or sleep. One parent attended the measurement. The stimuli were presented using two loudspeakers positioned at a distance of approximately 2 m in front of the baby and an angle of 30° from the infant’s head.

### 2.4 Data processing and analyses

FNIRS data were pre-processed using open source software HOMER2 which is implemented in MATLAB (Mathworks, Natick, MA) (Huppert et al., 2009). First, raw optical intensity data series (voltage) were converted into changes in optical density data. Then, channels with very high or very low optical density and channels with low signal to noise ratio were pruned from individual participants datasets. Next, a principal component analysis (PCA) was used to filter out motion artifacts. Components accounting for 95% of the covariance of the data were filtered out. To eliminate high-frequency instrument noise in the optical density data, the resulting time course was low-pass filtered with a cut-off frequency of 0.5 Hz. Next, changes in the concentration of oxyhemoglobin (HbO) and deoxyhemoglobin (HbR) were calculated from changes in optical density using the modified Beer-Lambert law with a partial pathlength factor for both wavelengths of 6.0. The hemodynamic response function was estimated by using a general linear model (GLM). The GLM was done using a series of Gaussian functions with a standard deviation of 1.0 s and their means separated by 1.0 s, and baseline drift was modeled with a 3rd order polynominal drift regressor (Huppert et al., 2009). We focused especially on HbO as this variable has been reported to be the strongest marker of neural responses in neonatal fNIRS (Gervain et al., 2011; Lloyd-Fox et al., 2010). Finally, for each individual participant changes in each of the 24 source-detector channels were exported for subsequent analysis by averaging across 20 s of each block starting 5 s post-stimulus onset, to account for the lag in hemodynamic response, until 25 s post-onset.

We statistically analyzed the data using SPSS Statistics 24 (IBM). As HbO data were normally distributed, a repeated measures ANOVA was conducted with within-subjects factors condition (language forward, language backward) and hemisphere (left, right) with the between-subjects factor group (preterm, full term) and for each group separately. Post-hoc analyses of main effects were calculated using paired t-tests and two sample t-tests. Significance is reported after Benjamini-Hochberg procedure to control for a false discovery rate of 15%. The value threshold was set at p_corr_ < 0.05. Furthermore, a Pearson correlation was calculated to examine the relationship between GA at birth and HbO response size within the group of preterm born infants.

Additionally, we performed a cluster-based permutation test on the hemodynamic response of the HbO signal to examine whether neonates respond differently to the two different language conditions across time and individual channels. Channels´ spatial neighborhood was defined as all channels within 1.5 cm. The t-score threshold for the cluster was +/- 2.36 (which corresponds to an uncorrected p-value of 5%). All 24 channels and time points until 25 s post-onset were included in the analysis.

## 3 Results

ANOVA with within-subjects factors condition and hemisphere and between-subjects factor group revealed a significant main effect of condition (*F*(128) = 7.635, p = .010, η^2^p = .214) and a significant interaction between condition and group (*F*(128) = 4.695, p = .039, η^2^p = .144). No significant main effect of hemisphere (*F*(128) = .558, p = .461, η^2^p = .020) nor a significant interaction between hemisphere and group was found (*F*(128) = 1.534, p = .225, η^2^p = .052).

**Fig. 2:**
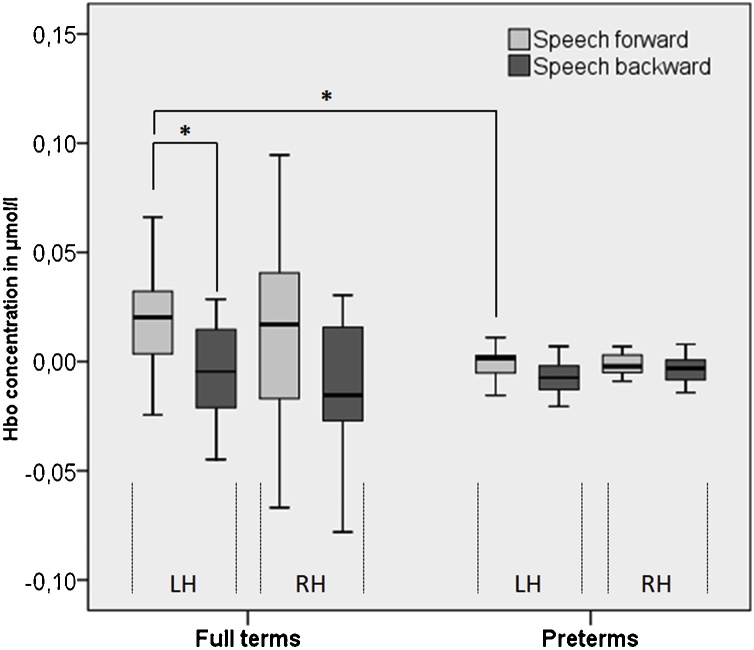
Mean group oxyhemoglobin (HbO) concentration of 24 channels during forward (light gray) and backward (dark gray) speech in preterm and full term infants. Full term neonates showed significantly more neural activation in response to forward speech compared to backward speech in the left hemisphere. Full term neonates activated left hemisphere regions during forward speech significantly more than preterm infants at term-equivalent age.

Post-hoc two sample t-tests exhibited a significant difference between the preterm and the full term group in the left hemisphere response to forward speech (full term: mean = .019, SD = .025; preterm: mean = -.002, SD = .009; t = 3.09, p = .006), whereas there was no significant difference between groups in hemodynamic response following forward speech in the right hemisphere (t = .28, p = .781) nor following backward speech in the left (t = 1.402, p = .181) or the right (t = -.67, p = .513) hemisphere (Fig. 2).

In the full term group, post-hoc paired *t*-test showed that in the left hemisphere, significantly more HbO increase was seen in reaction to forward (mean = .019, SD = .025) compared to backward speech (mean = -.005, SD = .021; t = 3.82, p = .002). No significant difference between forward and backward speech was found in the right hemisphere (t = 1.70, p = .112).

In the preterm group, post-hoc paired *t*-test revealed no significant difference between forward and backward speech, neither in the left (t = 1.73, p = .106) nor in the right hemisphere (t = 1.04, p = .702). We further explored if the preterm group did respond to auditory stimulation at all, irrespective of condition: One sample *t*-test showed a significant HbO signal change (t = 2.19, p = .046), especially in the left hemisphere (t = 2.31, p = .037).

In the preterm group, HbO responses to forward and backward speech, respectively, were not significantly correlated with GA at birth (all p > .05, r = from .13 to .20).

**Fig. 3:**
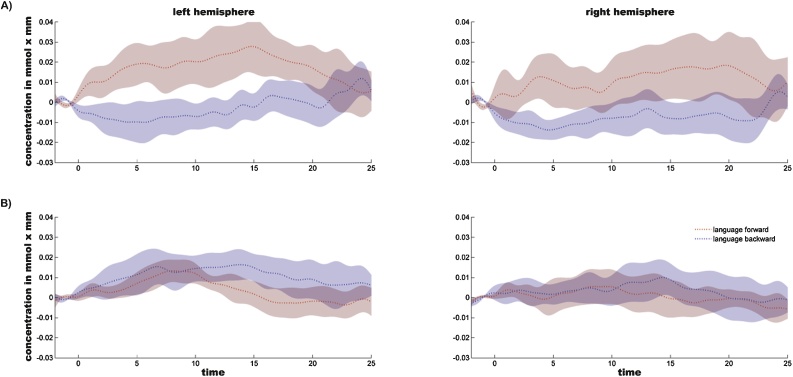
Mean time course of hemodynamic response to speech forward (red dotted lines) and speech backward (blue dotted lines) for the full term group (A) and the preterm group (B). Red and blue shades indicate the standard deviation. The x-axes display time in seconds, the y-axes represent concentration changes in μmol/l, averaged over all channels and all stimuli per condition (For interpretation of the references to colour in this figure legend, the reader is referred to the web version of this article).

Fig. 3 displays the time courses of hemodynamic responses within groups.

### 3.1 Cluster-based permutation analysis

**Fig. 4:**
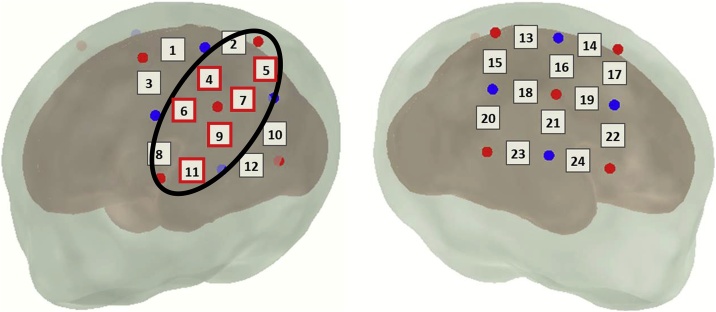
Cluster-based permutation analysis in full term neonates. A significant cluster was found in the left hemisphere including channels 4, 5, 6, 7, 9, and 11.

In the group of full term neonates, cluster-based permutation analysis revealed significantly greater changes in the HbO signal following forward speech compared to backward speech in full term neonates. A significant cluster (p = .028) was found in the left hemisphere including channels 4, 5, 6, 7, 9, and 11, covering inferior and middle frontal, central, superior temporal, and inferior parietal areas of the left hemisphere (Fig. 4). The reverse contrast, i.e. speech backward versus speech forward, revealed no significant cluster of channels.

In contrast, in the preterm group cluster-based permutation analysis did not reveal any significant cluster in the statistical comparison between the two conditions.

In sum, groups significantly differed in their hemodynamic responses to forward speech, whereas they showed no significant differences in their neural activations following backward speech. The full term group exhibited significantly more neural activation in response to forward speech compared to backward speech. This significance was due to differences between forward and backward speech processing within the left hemisphere, and cluster-based permutation analysis revealed a significant cluster extending from superior temporal over inferior parietal to middle and inferior frontal areas within the left hemisphere.

The preterm group signficantly responded to auditory stimulation, but did not show different neural activations in response to forward compared to backward speech.

## 4 Discussion

The present fNIRS study investigated speech discrimination in a group of preterm infants at term-equivalent age compared to full term neonates. Full term infants revealed significantly more hemodynamic response to forward compared to backward speech within the left hemisphere. In contrast, preterm infants did not show significant differences in their hemodynamic responses to forward versus backward speech suggesting they did not discriminate between speech and non-speech. In direct comparison with one another, the groups significantly differed in their task specific responses. These group differences were predominantly due to significant differences of responses to forward speech, whereas the groups did not differ in their responses to backward speech. These findings point to an altered development of the functional network underlying language acquisition in infants born preterm already at term-equivalent age.

### 4.1 Speech discrimination in full term neonates

In concordance with earlier studies our results demonstrate that when children are born at term, their brain is already specialized to respond to speech (Benavides-Varela and Gervain, 2017; Benavides-Varela et al., 2011; Gervain et al., 2008; May et al., 2011, 2018). Previous studies suggested that already at birth, recognizable language stimuli are preferentially processed in the left hemisphere, thus, left hemisphere regions may be activated by the recognition of familiar acoustic structures and even early verbal memory retrieval (Bortfeld, Shaw, & Depowski, 2013; Dehaene-Lambertz et al., 2002.; Vannasing et al., 2016).

Accordingly, the present study shows that full term neonates exhibit significantly different hemodynamic responses during speech compared to non-speech in the left hemisphere and is thus in line with previous studies using comparable cognitive paradigms of forward versus backward speech (Peña et al., 2003; Sato et al., 2012; Vannasing et al., 2016).

Cluster-based permutation analysis in the full term group revealed a significant cluster within the left hemisphere extending from superior temporal to inferior parietal and inferior and middle frontal areas. Unfortunately, an exact mapping of the optodes’ positions on the infants’ brains is not possible in the present study due to the lack of a co-registered structural MRI, which is a drawback of the study and has to be taken into account when interpreting these cluster analysis results. However, careful probe placement suggests that this significant cluster incorporates parts of the superior temporal gyrus, supramarginal gyrus, angular gyrus, and inferior and middle frontal gyrus. All these areas have been comprehensively proven to be essential language sites, in adults as well as in children, especially for processing phonology and lexico-semantics (Bartha-Doering et al., 2018b; Friederici and Gierhan, 2013; Price, 2012).

### 4.2 Speech discrimination in preterm infants

Infants born preterm did not significantly differentiate between forward and backward speech. Furthermore, compared to the full term group, preterm infants showed significantly less hemodynamic response during forward speech. In general, HbO signal changes were small in the preterm group, but they significantly responded to auditory stimuli, and the neural responses during backward speech were not significantly different between groups. These findings thus point toward a normal basic acoustic and phonemic processing in preterm infants. However, the lack of differences in hemodynamic responses between forward and backward speech may indicate reduced speech discrimination abilities in preterm infants, and the differences in preterms compared to full terms in neural activations following forward speech point to an altered language network in preterm born infants already at term-equivalent age.

Our findings add to the study of Arimitsu et al. (2018). They described atypical patterns of hemodynamic responses to phonetic and prosodic changes of syllables in preterm born neonates at the GA of 33–38 weeks which diminished when preterm born infants reached term age. The present study could also find increases in hemodynamic responses in the preterm group in response to auditory stimuli, confirming in part the findings of Arimitsu et al. (2018). However, these increases in hemodynamic responses in our preterm group did not statistically differ with regard to stimulus type, thus, the preterms did not show significant neural discrimination between speech and non-speech at term. This finding is different to that by Arimitsu et al. (2018) who showed a normalization of previous atypical hemodynamic responses at 39–41 weeks of GA. These divergent findings between Arimitsu et al.’s study and ours may have several reasons. First, the paradigms in these two studies differ considerably. Arimitsu et al. (2018) used three forms of a Japanese word, /itta/, /itte/ and itta?/, which were contrasted against /itta/. We presented sentences of spoken language in sequences of 15 s with well-formed and complete prosodic units in contrast with the same sequences reversed, which shared the acoustic and phonetic features, but differed in phonological, semantic, and prosodic information. It may be hypothesized that our paradigm requires a more complex, linguistically demanding auditory differentiation compared to Arimitsu et al.’s paradigm, and that we can thus find more subtle differences in preterm born neonates at term-equivalent age. Second, Arimitsu et al. (2018) used a categorial classification of typicality of hemodynamic responses, with *typicality* defined as a positive correlation between the canonical hemodynamic response function model and the time course of hemoglobin changes. Gradual increases or decreases of hemoglobin changes are thus not recognized in this analysis, as long as the time course of hemoglobin changes represents the typical hemodynamic response function pattern. In contrast, we quantitatively investigated intra- and between-group differences of hemodynamic responses by using repeated measures ANOVA and cluster-based permutation analysis. These significantly different analysis strategies may also explain the different findings in Arimitsu et al.’s study and ours. Third, the atypical response and lateralization pattern of preterm born infants with GA of 33–38 described by Arimitsu et al. (2018) may be influenced by differences in head circumferences. Unfortunately, Arimitsu et al. (2018) did not report group results and possible group differences of head circumferences. It may be hypothesized that infants with younger GA had smaller head circumferences which may have influenced the position of the optodes. Thus, the channels may not have been measuring the same areas and groups may not have been precisely comparable. Nevertheless, the study of Arimitsu et al. (2018) together with the present results point to differences in neural speech processing in preterm born infants.

One reason for the differences in neural speech discrimination between our preterm and full term infants might be the very early, intrauterine auditory experience in fetuses. The cochlea reaches adult size at as early as 21 weeks of GA, and the auditory systems starts being functional at around 25 weeks of GA (Lim and Brichta, 2016; Mejdoubi et al., 2016). By this age, peripheral auditory input reaches the auditory cortex, and auditory memory traces start to develop (Mahmoudzadeh et al., 2013; Partanen et al., 2013). The fetus receives speech in utero low-pass filtered by maternal tissue (Benavides-Varela and Gervain, 2017; Querleu et al., 1988). Therefore, full term neonates have auditory experience with speech before delivery.

Accordingly, preterm children are born during a critical period for the development of the auditory cortex. Very recently, Monson et al. (2018) have shown that gray and white matter maturation of the auditory cortex is disturbed by preterm birth, and that the disturbance of non-primary auditory cortex is associated with delayed language development at the age of two years. Early auditory experience, either in form of auditory enrichment or auditory deprivation, can have substantial impact on the structural and functional development of the auditory cortex (Chang and Merzenich, 2003; Harshaw and Lickliter, 2011). Webb et al. (2015) showed that the preterm infant’s brain is shaped by exposure to maternal sounds in the first weeks of life und highlighted the importance of auditory experience before maturity. The authors examined the thickness of the auditory cortex in preterm infants receiving auditory enrichment via audio recordings of their mother’s sounds versus routine exposure to hospital environmental noise. They found a significantly larger auditory cortex bilaterally in those children who received their mother’s sounds and thus revealed that the preterm auditory cortex is more adaptive to maternal sounds than environmental noise.

In the present study, preterm infants were investigated around 40 weeks of GA in order to have the same gestational age as the full term group. Thus, preterm babies staying at the neonatal care unit experienced completely different auditory inputs in the weeks prior to the measurement compared to full term neonates who were delivered a few days before the measurement and had a longer auditory experience in their mother’s womb. This may have influenced the infants’ neural speech perception in the present study.

These findings may furthermore add to the ongoing clinical discussion of environmental modifications to a special care environment at the neonatal intensive care unit. In their systematical review, Best et al. (2018) have outlined that preterm born infants experience low levels of language exposure and high levels of sound exposures in neonatal intensive care units. Our findings together with previous research showing facilitation of auditory cortex development through auditory enrichment (Chang and Merzenich, 2003; Harshaw and Lickliter, 2011) emphasize the relevance of early language exposure after preterm birth and provide further evidence for the use of language as an important intervention to enhance neurodevelopment in preterm born children (Best et al., 2018).

We did not find a significant correlation of GA at birth with hemodynamic signal change within the group of preterm born children, thus, the degree of preterm birth did not significantly influence speech discrimination in our participants. It may be hypothesized that primary and secondary auditory cortices may not develop linearly with age, but need to reach a critical stage of intrauterine development. However, future research is needed with larger samples to investigate if and how the degree of preterm birth relates to speech discrimination.

### 4.3 Reasons for preterm birth and altered functional brain development

However, the cognitive differences between preterm and full term neonates may not only be due to the interruption of an otherwise normal prenatal development. The same reasons for preterm birth may additionally underlie altered functional brain development both pre- and postnatal, at least in some children. Endocrine, immune, vascular, and genetic mechanisms may not only influence preterm parturition, but also functional brain development (Adams Waldorf and McAdams, 2013; Miranda and Sousa, 2018). Thomason et al. (2017) have used resting state functioning magnetic resonance imaging in high-risk-for-early-delivery-fetuses subsequently born preterm and found reduced neural connectivity within language associated regions already before birth. Thus, the speech discrimination deficits in preterm infants found in the present study may probably be due to a combination of biological, genetic, and environmental factors influencing functional brain development both in utero and postnatally.

### 4.4 Linguistic and non-linguistic cognitive functions in preterm born children

The present study shows that preterm infants at term-equivalent age do not process backward speech significantly differently from full term neonates; thus, basic acoustic and phonemic processing may be present in preterm infants. However, neural differentiation of forward and backward speech was not existent in the preterm group, pointing to an early, specific deficit of linguistic processing. This may suggest an independence of linguistic deficits from general cognitive functioning in children born preterm. There is controversy in the literature regarding the role of different cognitive skills in language development of children born preterm. Cognitive domains are not isolated in their developmental trajectories suggesting that delays within one cognitive domain influence the development of others (Karmiloff-Smith, 1998). Accordingly, interactions between working memory, fluid intelligence, and attention with language can be observed during development (Engel de Abreu et al., 2010, 2011). Studies in preterm children have shown contrasting results. Some studies found persisting linguistic deficits after they controlled for non-linguistic cognitive scores and interpreted their findings as a primary linguistic impairment underlying the language deficits in preterm children (Guarini et al., 2009, 2013). Others could statistically explain the language deficits in preterm children by differences in other cognitive domains and suggested a general cognitive delay after preterm birth (Ortiz-Mantilla et al., 2008; Ribeiro et al., 2011; Rose et al., 2009; Wolke et al., 2008). A recent study by Guarini et al. (2016) comprehensively investigated language skills in preterm children at preschool age and showed that the relationship between language and other cognitive skills may be task dependent: whereas grammatical and phonological skills were significantly associated with nonverbal cognitive functioning, deficient lexical skills such as reduced expressive vocabulary and auditory word comprehension were independent of general cognitive functioning. This is in line with the findings of the present study, suggesting normal acoustic and phonemic processing but altered neural speech discrimination in preterm infants already at term-equivalent age.

### 4.5 How do these findings inform our understanding of very early language development?

Several studies prove an association between language localization and language functioning in children, though the exact relationship is far from being clear (Bartha-Doering et al., 2018a, b; Bartha-Doering et al., 2019; Berl et al., 2010; Sepeta et al., 2016). Previous studies in preterm born adolescents showed altered functional language networks associated with language deficits (Northam et al., 2012; Salvan et al., 2017). The present study demonstrates that the altered language network in preterm children is not only the effect of reduced language acquisition and processing during childhood, but is already present shortly after birth and associated with altered neural speech discrimination. These findings suggest that the time between the last prenatal trimester and the first postnatal days is a vulnerable phase for language development, and disturbances during this time may result in an altered language network.

## 5 Conclusion and outlook

This study exhibits altered neural speech discrimination in preterm born infants at term-equivalent age und thus forms the basis for future research. An association of very early neural speech discrimination and later language development would evidence speech discrimination in the neonatal stage as an early marker for later language deficits. Recognizing early neurobiological markers of later language deficits in single individuals would help to predict who is at risk for language impairment and could improve planning of therapy strategies.

Our results furthermore underline previous studies on special care environment at the neonatal intensive care unit that emphasize the relevance of early language exposure after preterm birth to enhance neurodevelopment in preterm born children. An auditory environment at the neonatal care unit closely comparable to in utero, including parental voices and reduction of environmental sounds, may support the auditory cortex development of preterm born children and thus facilitate language development.

## Funding

This work was supported by the Austrian Science Fund (FWF), Project number KLI544-B27.

## Declaration of Competing Interest

The authors declare that they have no known competing financial interests or personal relationships that could have appeared to influence the work reported in this paper.
